# H3 Relaxin Alleviates Migration, Apoptosis and Pyroptosis Through P2X7R-Mediated Nucleotide Binding Oligomerization Domain-Like Receptor Protein 3 Inflammasome Activation in Retinopathy Induced by Hyperglycemia

**DOI:** 10.3389/fphar.2020.603689

**Published:** 2020-12-16

**Authors:** Kelaier Yang, Jiannan Liu, Xiaohui Zhang, Ziqi Ren, Lei Gao, Ying Wang, Wenjian Lin, Xuefei Ma, Ming Hao, Hongyu Kuang

**Affiliations:** ^1^ The Department of Endocrinology, The First Affiliated Hospital of Harbin Medical University, Harbin, China; ^2^ The Department of Urology, The First Affiliated Hospital of Harbin Medical University, Harbin, China; ^3^ The Department of Cardiology, The First Affiliated Hospital of Harbin Medical University, Harbin, China

**Keywords:** relaxin, NLRP3 inflammasome, diabetic retinopathy, P2X7, diabetes

## Abstract

**Introduction:** P2X7R excitation-interrelated NLRP3 inflammasome activation induced by high glucose contributes to the pathogenesis of diabetic retinopathy (DR). Relaxin-3 is a bioactive peptide with a structure similar to insulin, which has been reported to be effective in diabetic cardiomyopathy models *in vivo* and *in vitro*. However, it is not known whether relaxin-3 has a beneficial impact on DR, and the underlying mechanisms of the effect are also remain unknown.

**Methods and Results: **The retinas of male streptozotocin (STZ)-induced diabetic Sprague-Dawley (SD) rats were characterized. Human retinal microvascular endothelial cells (HRMECs) were used to evaluate the anti-inflammatory, antiapoptotic, antipyroptotic and anti-migration effects of H3 relaxin by transmission electron microscopy, wound-healing assay, transwell assay, flow cytometry, cytokine assays and western-blot analysis. After H3 relaxin treatment, changes of the ultrastructure and expression of NLRP3 inflammasome related proteins in the retinas of rats were compared with those in the diabetic group. *In vitro*, H3 relaxin played a beneficial role that decreased cell inflammation, apoptosis, pyroptosis and migration stimulated by advanced glycation end products (AGEs). Moreover, inhibition of P2X7R and NLRP3 inflammasome activation decreased NLRP3 inflammasome-mediated injury that similar to the effects of H3 relaxin. H3 relaxin suppressed the stimulation of apoptosis, pyroptosis and migration of HRMECs in response to AGEs mediated by P2X7R activation of the NLRP3 inflammasome.

**Conclusion:** Our findings provide new insights into the mechanisms of the inhibitory effect of H3 relaxin on AGE-induced retinal injury, including migration, apoptosis and pyroptosis, mediated by P2X7R-dependent activation of the NLRP3 inflammasome in HRMECs.

## Introduction

Diabetic retinopathy (DR) is a predominant comorbidity caused by uncontrolled hyperglycemia that leads to severe visual impairment and blindness (The Diabetes Control and Complications Trial Research Group, 1993; Yau et al., 2012). Sustained hyperglycemia, which impacts the homeostasis of the retinal vasculature and the integrity of the blood-retinal barrier (BRB), is regarded as the dominant promoter of DR development (Perrone et al., 2014; Simó and Hernández, 2014; Kowluru et al., 2015). Retinal microvessels are the pervasive targets of diabetic damage, which is characterized by inflammation, neovascularization, and abnormal permeability of the BRB. During hyperglycemia, dysfunction of retinal microvascular endothelial celsl (RMECs) is considered to be the initiator of a multifactorial etiology and pathogenesis, which are manifested as close relationships with cell migration and apoptosis upon exposure of cells to the cellular advanced glycation end products (AGEs) and are regulated by a variety of inflammatory and apoptotic factors (Mizutani et al., 1996; Stitt et al., 2016). Identification of innovative approaches for the prevention and treatment of DR is essential for neutralization of the negative consequence of vision loss related to hyperglycemia.

Nucleotide binding oligomerization domain-like receptor protein 3 (NLRP3) inflammasome, a kind of intracellular supramolecular protein complex composed of the core sensor protein NLRP3, the adaptor apoptosis-associated speck-like protein containing a CARD (ASC) and effector caspase-1 (Duncan et al., 2007). Upon stimulation, ASC interacts with the upstream NLRP3 protein and recruits the downstream target caspase-1. Caspase-1 is activated by autoproteolysis to form the active p10 and p20 subunits. Active caspase-1 mainly acts on interleukin (IL)-1 family substrates, promoting the release of IL-1β and IL-18 (Schmidt et al., 2016; Willingham et al., 2009; von Moltke et al., 2013). Several models of NLRP3 inflammasome activation have been developed, including an ion redistribution model, a lysosomal disruption model, a mitochondrial dysfunction model, a metabolic changes model and a trans-Golgi disassembly model. The ion redistribution model mainly includes potassium ion (K^+^) efflux, calcium ion (Ca^2+^) flux and chloride ion (Cl^−^) efflux. The K^+^ ionophore and ATP-mediated purinergic ligand-gated ion channel seven receptor (P2X7R), a ligand-gated ion channel in the purinergic receptor family, promote the secretion of IL-1β and IL-18 via K^+^ efflux and Ca^2+^ influx (Surprenant et al., 1996; Samways et al., 2014). P2X7R and the NLRP3 inflammasome always intimately interact and colocalize at discrete spots in the subplasmalemmal area in the resting and active states. The NLRP3 inflammasome is the primary transducer that translates a decrease in cytoplasmic K^+^ induced by P2X7R receptor activation into pro-inflammatory signals (Franceschini et al., 2015). P2X7R has various polymorphic variants with variable abilities to increase cytosolic Ca^2+^ levels upon induction by ATP. Treatment with P2X7R antagonists and data obtained from P2X7^−/−^ mice demonstrates that suppression of P2X7R attenuates inflammatory processes, indicating that P2X7R plays the role of a trigger that decreases intracellular K^+^, and is an essential ATP-sensing receiver of NLRP3 inflammasome signaling (Karmakar et al., 2016). Therefore, inhibition of P2X7R expression indirectly reduces the activation of the NLRP3 inflammasome.

P2X7R is considered as a potential pharmacological target in several ocular diseases (Kerur et al., 2013; Beckel et al., 2014; Sanderson et al., 2014). Validated selective P2X7R antagonists are currently being evaluated in Phase I/II clinical trials (Clapp et al., 2019). P2X7R plays an important role in BRB integrity by regulating trans-endothelial electrical resistance (TEER) and the cell junction morphology (Platania et al., 2019). P2X7R antagonists protect against alterations of mitochondrial function and changes in cell permeability, which are caused by enhanced vulnerability to P2X7R activation in retinal microvessels; these properties of P2X7R antagonists suggest their potential use in the treatment of DR (Platania et al., 2017). Active P2X7R signaling induces vasotoxicity mediated by the activation of K^+^ and Ca^2+^ channels, as well as ROS accumulation in retinal pericytes and capillaries to potentially amplify the inflammatory reaction induced by high glucose (Shibata et al., 2018).

Relaxin is a multipotent peptide hormone of the insulin superfamily that was discovered in the circulating blood of pregnant women in 1926 (Bathgate et al., 2002). Relaxin plays an anti-inflammatory role in inflammatory response by enhancing the synthesis of endogenous nitric oxide and inhibiting the production of inflammatory mediators such as IL-1, IL-6 and tumor necrosis factor-α (TNF-α) (Masini et al., 2004; Bani, 2012). It should be noted that relaxin can improve insulin resistance, enhance pancreatic beta cell function, restore vascular endothelial dysfunction and promote the remediation of injured tissue under diabetic conditions (Szepietowska et al., 2008). Previous studies on the relaxin family have mainly focused on relaxin-2, the biological role of relaxin-3 is similar to that of relaxin-2. Relaxin-3 is present in the brain and participates in the regulation of feeding, learning, memory and physiological stress (Bathgate et al., 2002). It has been confirmed that H3 relaxin (synthetic relaxin-3) inhibits the apoptosis and fibrosis in ventricular myocytes induced by high glucose through regulating both external and internal pathways (Zhang et al., 2005). Similarly, our previous studies have shown that H3 relaxin can alleviate inflammation and fibrosis in the kidneys of diabetic rats, however, it is not known whether H3 relaxin is effective against diabetic retinopathy, which belongs to the group of microvascular complications of diabetes along with diabetic nephropathy, is still unknown.

Our study aimed to evaluate the anti-inflammatory, antiapoptotic and anti-cell migration effects of H3 relaxin and to determine whether H3 relaxin inhibits cell migration and apoptosis through the regulation of P2X7R-mediated activation of the NLRP3 inflammasome.

## Materials and Methods

### Experimental Animals and Groups

A total of 110 adult male Sprague-Dawley (SD) rats (weighing 200–250 g) were obtained from the Experimental Animal Centre at the Second Affiliated Hospital of Harbin Medical University. The rats were randomly divided into control (Group C, n = 30) and diabetic groups after stable feeding. The animals in the diabetic group was established by intraperitoneal injection of 65 mg/kg STZ (Sigma, St. Louis, MO, USA; diluted in 0.1% mol/L citrate buffer, pH = 4.5). After successful development of the diabetic model, the diabetic group was randomly divided into three subgroups containing approximately equal number of animals (n = 26–29): a high-dose H3 relaxin group (2 µg/kg/d, Group A), a low-dose H3 relaxin group (0.2 µg/kg/d, Group B) and a diabetic group treated with the same dose of normal saline (Group D). Synthetic H3 relaxin (Cat#035–36A, Phoenix Pharmaceuticals, USA; diluted in sterile water for injection) was injected into Group A and Group B rats for 14 consecutive days during the last 2 weeks before the end of the experiment. The retinas and plasma were obtained after 4 and 8 weeks of STZ treatment, and the samples were fixed or stored at specific temperature for subsequent imaging or biochemical analyses.

The study was approved by the Ethics Committee of The First Affiliated Hospital of Harbin Medical University. All animal studies and experimental protocols were carried out in accordance with the Animal Management Rule of the Ministry of Health in the People’s Republic of China (Document No. 55, 2001) and the Guide for the Care and Use of Laboratory Animals published by the US National Institutes of Health (NIH Publication No. 85-23, revised 1996).

### Human Retinal Microvascular Endothelial Cell Culture

Human retinal microvascular endothelial cells (hRMECs) were obtained from the Ophthalmology Laboratory of Harbin Medical University and cultured in endothelial cell medium (ECM; Sciencell, San Diego, CA, United States) supplemented with 5% fetal bovine serum (Sciencell, San Diego, CA, United States), 100 U/ml penicillin and 100 µg/ml streptomycin in a humidified atmosphere of 5% CO2 at 37°C. hRMECs were used in the experiments at passages 3–6. The cell experiments involved three stages. In the first phase, the cells were pretreated with H3 relaxin (100 ng/ml), insulin (50 nmol/L, Novo Nordisk), both or physiological saline for 30 min with exposure to AGE-BSA (100 µg/ml, Cat#2221-10, BioVision, San Francisco, CA, USA) or control-BSA (100 µg/ml, BioVision, San Francisco, CA, USA) for 48 h. In the second phase, the cells were treated with H3 relaxin (100 ng/ml), MCC950 (1 µmol/L, Cat#PZ0280, Sigma-Aldrich, St. Louis, MO, USA), both or physiological saline with exposure to AGE-BSA (100 µg/ml) or control-BSA (100 µg/ml) for 48 h. In the third phase, cells were treated with control-BSA (100 µg/ml), AGE-BSA (100 µg/ml), AGE-BSA+H3 relaxin (100 ng/ml), AGE-BSA+BzATP (100 µmol/L, Cat#B6396, Sigma-Aldrich, St. Louis, MO, United States), AGE-BSA+A438079 (10 µmol/L, Cat#A9736, Sigma-Aldrich, St. Louis, MO, United States), or AGE-BSA + BzATP (100 µmol/L)+ H3 relaxin (100 ng/ml) for 48 h.

### Observation of the Ultrastructure by Transmission Electron Microscopy

To prepare retinal samples, after anesthetizing and sacrificing the rats, the whole eyeball was immediately extracted and cutted into hemisles by the optic nerve, then fixed the samples in 2.5% pre-cooling glutaraldehyde. To prepare hRMECs samples for the observation of apoptosis and pyroptosis, cells were treated according to experimental requirements, digested with the enzyme trypsin, centrifuged at 3,000 rpm for 15 min and fixed in 2.5% pre-cooling glutaraldehyde.

After at least 2 h of fixation under the condition of 4°C, the samples were further fixed with 2% cacodylate-buffered 2% osmium tetroxide, followed by dehydrating in an ascending alcohol procedure and embedding in Epon 812. Then, ultrathin sections which are about 50 nm thick were cutted and loaded on the copper net. After staining the sections with uranyl acetate and lead citrate, the samples were observed under a transmission electron microscope (JEM 1210; JEOL, Ltd., Japan).

### Measurement of Inflammatory Factor Levels in the Plasma

After anesthetization, plasma samples were obtained from the abdominal aorta of rats and then were centrifuged at 1,000 rpm for 10 min. The plasma samples were used to detect IL-18, IL-1β, vascular endothelial growth factor (VEGF) and TNF-α concentrations with a Cytokine/Chemokine Magnetic Bead Panel kit (Cat#RECYTMAG-65K, EMD Millipore, Germany).

### Detection of Cytokines in the Culture Medium

Culture medium was extracted by centrifuging at 1,000 g for 10 min, and IL-18 (Cat#EK0864, Bosterbio, Wuhan, China) and IL-1β (Cat#EK0392, Bosterbio, Wuhan, China) concentrations were measured by enzyme-linked immunosorbent assay.

### Western Blot Analysis

Protein was harvested from rat retinal tissue and hRMECs using RIPA buffer with protease inhibitors, and the protein concentration was determined by the BCA protein assay (Beyotime, China). Protein samples were separated with 10% and 12% SDS–PAGE in a running buffer and transferred to PVDF membranes with a transfer buffer. After blocking in 5% skim milk, the membranes were incubated with the primary antibodies anti-NLRP3 (1:1,000, Cat#ab214185, Abcam), anti-ASC (1:500, Cat#sc-514414, Santa Cruz Biotech), anti-cleaved caspase-1 (1:1,000, Cat#4199, Cell Signaling Technology; 1:1,000, Cat#ab179515, Abcam), anti-IL-1β (1:500, Cat#sc-515598, Santa Cruz Biotech), anti-IL-18 (1:1,000, Cat#ab191152, Abcam; 1:500, Cat#bs-0529R, Bioss), anti-MMP-2 (1:1,000, Cat#ab92536, Abcam), anti-MMP-9 (1:1,000, Cat#ab76003, Abcam), anti-VE-cadherin (1:1,000, Cat#ab119785, Abcam), anti-ZO-1 (1:1,000, Cat#ab96587, Abcam; 1:2000, Cat#21773-1-AP, ProteinTech), anti-occludin (1:1,000, Cat#ab216327, Abcam), anti-cleaved caspase-3 (1:1,000, Cat#9661, Cell Signaling Technology), anti-cleaved caspase-9 (1:1,000, Cat#9508, Cell Signaling Technology; 1:1,000, Cat#ab2324, Abcam), anti-cleaved caspase-8 (1:1,000, Cat#13423-1-AP, ProteinTech), anti-P2X7R (1:1,000, Cat#ab48871, Abcam), anti-gasdermin D (1:1,000, Cat#39754, Cell Signaling Technology) and anti-β-actin (1:1,000, Cat#TA-09, ZSJQ-BIO), followed by incubation with peroxidase-conjugated secondary antibodies (1:1,000, Cat#zb-2305&zb-2301, ZSGB-BIO). The immunoreactive complexes were analyzed using Bio-Rad Image Lab software for quantification. All experiments were repeated at least three times.

### Wound-Healing Assay

After growing to complete confluency, hRMECs were treated with 10 μg/ml mitomycin C for 2 h. Wounds were created by scratching with a pipette tip, and then the cells were incubated according to experimental requirements for 24 h. Images of the wound areas were obtained at 0 and 24 h using a microscope to analyze cell movement. ImageJ software (National Institutes of Health, USA) was used to analyze the extent of migration. Each assay was repeated more than three times. The extent of wound healing was calculated as a percentage according to the following equation: [1−(empty area 24 h/empty area 0 h)]×100%.

### Transwell Assay

The migration of hRMECs was assessed with transwell chambers (Corning, United States). Pretreated cells were suspended in serum-free medium and then seeded in the upper compartment of the transwell chambers. The lower compartment was filled with medium containing 20% FBS. After a 24-h incubation, migrated cells were fixed with 4% paraformaldehyde and stained with 0.5% crystal violet. The degree of migration was analyzed under a light microscope, and the average number of migrating cells was calculated based on three replicate experiments.

### Flow Cytometry Analysis of Cell Apoptosis

FITC-conjugated Annexin V (Annexin V-FITC)/propidium iodide (PI) Apoptosis Detection kit was purchased from Beyotime Biotechnology (Shanghai, China). After treatment and centrifugation at 2000 rpm for 5 min under the condition of 4°C, hRMECs were harvested and added with binding buffer to re-suspend, followed by staining in the dark with Annexin V-FITC and PI for 15 min. A FACSCalibur flow cytometer (BD Biosciences, Franklin Lakes, NJ, United States) was employed to detect and analyze the apoptosis of hRMECs. The experiments were repeated three times.

### Statistical Analyses

Data are expressed as the mean ± SD, SPSS 22.0 was used to analyze the results. One-way ANOVA followed by the Newman-Keuls multiple-comparison test was used for statistical analysis of more than two groups. *p* < 0.05 was considered statistically significant.

## Results

### H3 Relaxin Improved the Ultrastructure of Rat Retinas Under High-Glucose Conditions

Transmission electron microscope was used to observe the layered structure of the retina in rats after 4 or 8 weeks of treatment. At 8 weeks, the retinal layers of the control group were intact and clear. After STZ treatment, disorganized membrane discs were observed, mitochondria in several layers were degenerated, and the numbers of synapses and synaptic vesicles in the inner plexiform layer and outer plexiform layer were decreased. The ganglion cell layer showed ganglion nuclei swelling and endoplasmic reticulum expansion. After administration of a high dose of H3 relaxin, the membrane discs were substantially clearer, the numbers of synapses in the outer and inner plexiform layers were increased in H3 relaxin-treated rats compared with the diabetic rats, and the morphology of the ganglion cells was normal; however, the improvement was less pronounced in the low-dose treatment group (Supplementary Figure S1). The degree of injury at 4 weeks was weaker than that at 8 weeks, however, the trends in each group were similar.

### H3 Relaxin Alleviated Inflammatory Activity in Diabetic Rat Retinas

**FIGURE 1:**
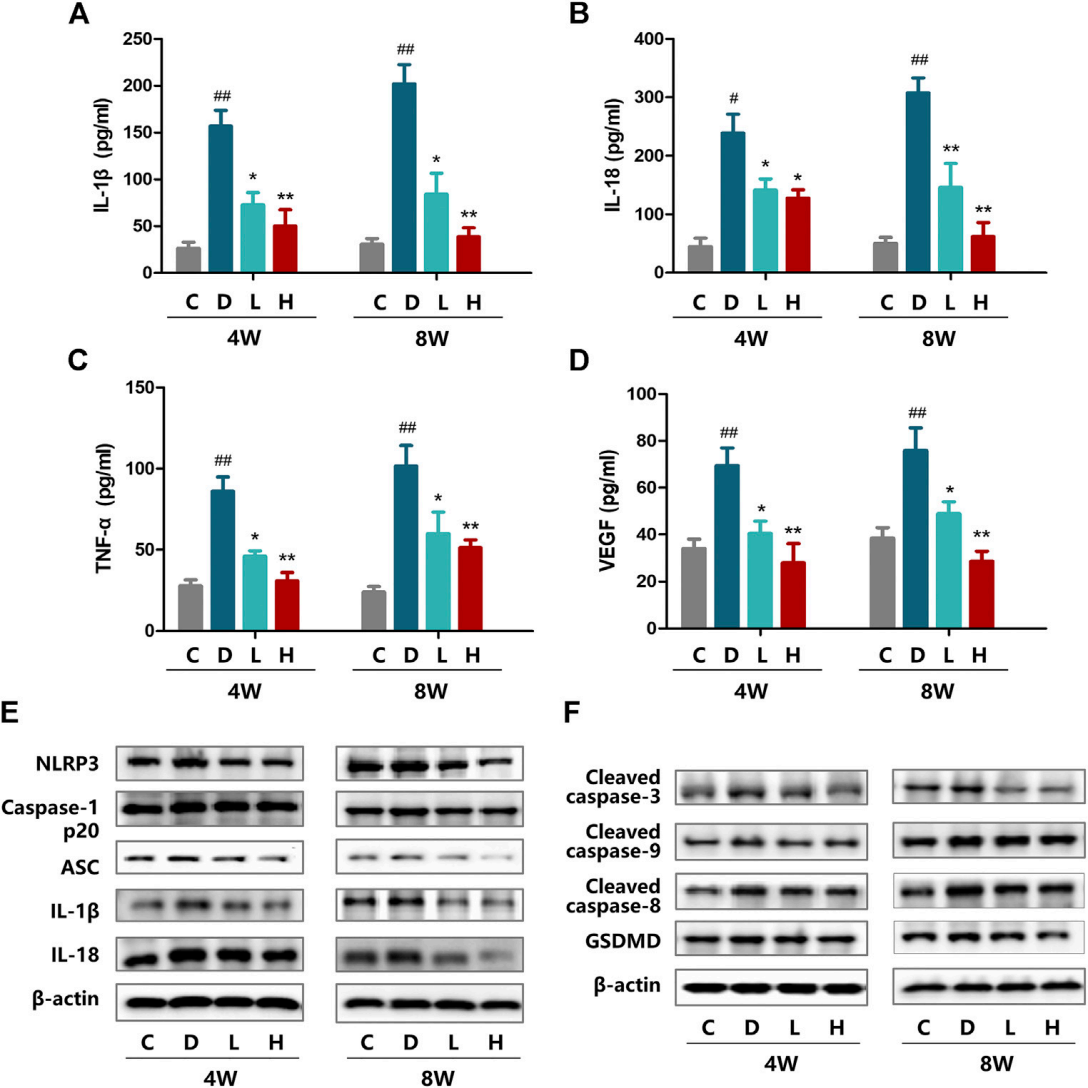
Effect of H3 relaxin treatment on inflammation and apoptosis in retinas of diabetic rats. **(A)** The expression of IL-1β at 4 and 8 weeks in the plasma of diabetic rats. **(B)** The expression of IL-18 at 4 and 8 weeks in the plasma of diabetic rats. **(C)** The expression of TNF-α at 4 and 8 weeks in the plasma of diabetic rats. **(D)** The expression of VEGF at 4 and 8 weeks in the plasma of diabetic rats. **(E)** The protein expression of NLRP3 inflammasome and inflammatory cytolines at 4 and 8 weeks were evaluated by western blot. **(F)** The expression of apoptosis-related protein at 4 and 8 weeks were evaluated by western blot. Data are the means ± SD, and each measurement was repeated six times. ^#^ *p* < 0.05 vs. Control group, ^##^ *p* < 0.01 vs. Control group, *p < 0.05 vs. Diabetic group, **p < 0.01 vs. Diabetic group.

To investigate whether inflammation is induced by hyperglycemia, the expression of the NLRP3 inflammasome and inflammatory cytokines IL-18 and IL-1β were assayed by western blot analysis. After STZ treatment, the levels of NLRP3, ASC, cleaved caspase-1, IL-18 and IL-1β were significantly increased at both 4 and 8 weeks. At 8 weeks, the expression levels of these components were higher than at 4 weeks (Figure 1E and Supplementary Figure SF2A,B). After administration of H3 relaxin, the expression of all the tested proteins decreased in the high-dose group compared with those in the low-dose group. Similar patterns were detected at the two time points at 4 and 8 weeks. Moreover, according to the cytokine kit analysis, the VEGF, TNF-α, IL-18 and IL-1β levels in rat plasma demonstrated similar trends, which also supported that H3 relaxin lessened the inflammatory activity induced by hyperglycemia (Figures 1A–D).

### H3 Relaxin Suppressed Apoptosis in the Rat Retina Under Diabetic Conditions

Western blot analysis was performed to evaluate the induction of apoptosis by hyperglycemia, including the death receptor and mitochondrial pathways characterized by the expression of caspase-8 and caspase-9, respectively, which were evaluated as well as the downstream executor caspase-3. The results indicated that the expression of cleaved caspase-3, cleaved caspase-8 and cleaved caspase-9 was enhanced in the high-glucose environment and that the levels of these proteins at 8 weeks were higher than those at 4 weeks. H3 relaxin reduced the expression of all of these proteins, and the high-dose treatment had a preferable protective effect than the low-dose treatment (Figure 1F and Supplementary Figure S2C,D).

### H3 Relaxin Weakened AGE-Induced NLRP3 Inflammasome Expression in hRMECs

**FIGURE 2:**
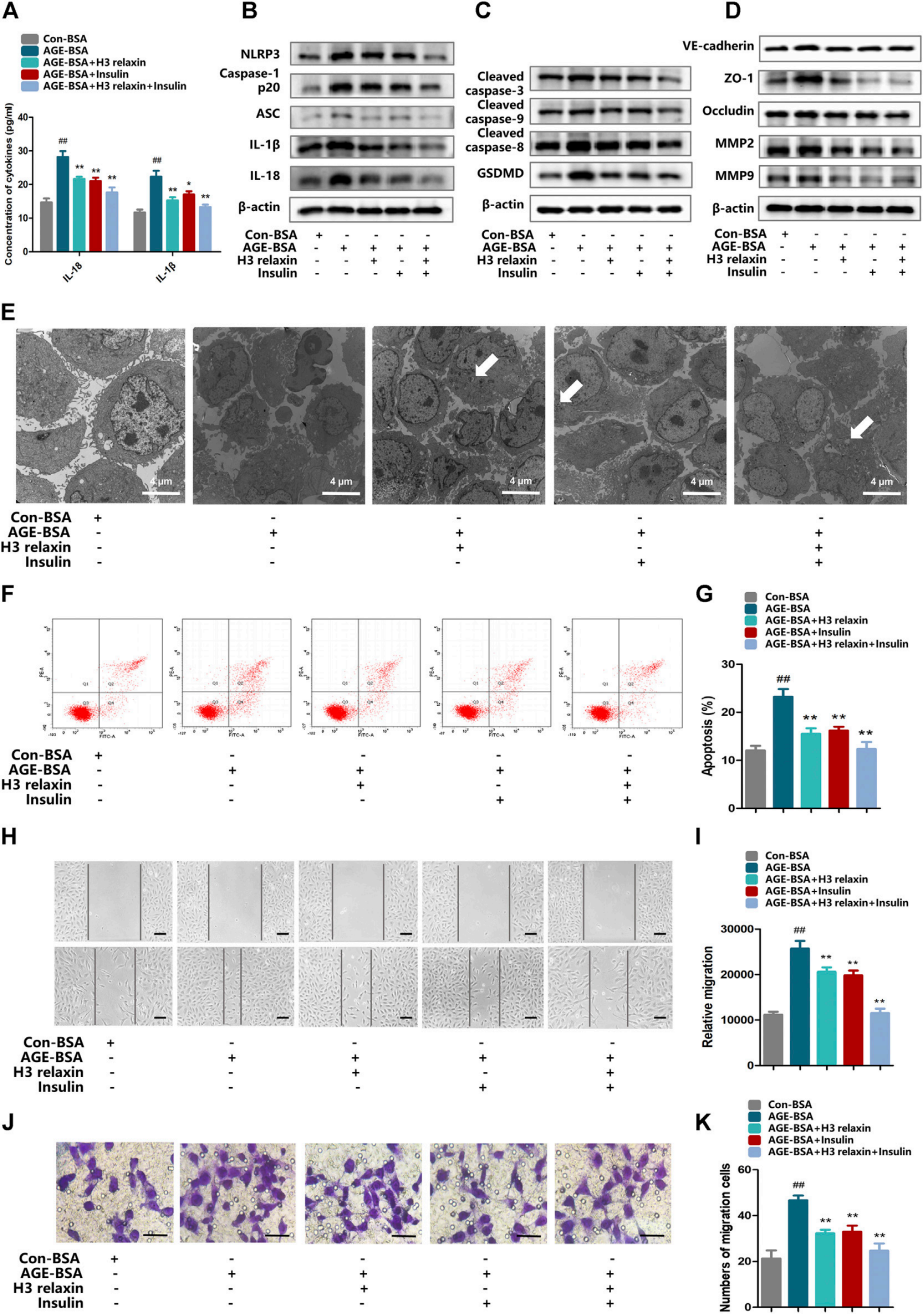
H3 relaxin alleviated NLRP3 inflammasome activation, apoptosis, pyroptosis and migration in hRMECs. **(A)** The protein levels of IL-1β and IL-18 in cell culture media. **(B)** The protein expression of NLRP3 inflammasome and inflammatory cytolines were evaluated by western blot. **(C)** The protein expression of cleaved caspase-3, cleaved caspase-9, cleaved caspase-8 and GSDMD were evaluated by western blot. **(D)** The protein expression of VE-cadherin, ZO-1, occludin, MMP2 and MMP9 were evaluated by western blot. **(E)** The apoptosis in hRMECs were observed by transmission electron microscopy. (original magnification, ×5,000). Ultrastructural evaluation was performed: The majority of hRMECs treated by AGE-BSA showed apoptosis in early and middle phase. After H3 relaxin or/and insulin treatment, hRMECs were noticed improvement in early phase of apoptosis. Medullary corpuscles (arrow) can be noticed. **(F,G)** Cell apoptosis was measured by flow cytometry. **(H,I)** Wound-healing assay of hRMECs (Scale bar = 200 µm). **(J,K)** Transwell assay of hRMECs (Scale bar = 200 µm). Data are the means ± SD, and each measurement was repeated six times. ^##^ *p* < 0.01 vs. Control group, *p < 0.05 vs. Diabetic group, **p < 0.01 vs. Diabetic group.

The expression of the NLRP3 inflammasome and relevant cytokines IL-18 and IL-1β was assayed in hRMECs. Compared with the con-BSA group, the AGE-BSA group showed heightened expression of NLRP3, ASC, cleaved caspase-1, IL-18 and IL-1β. Additionally, we observed protection by treatment with H3 relaxin, insulin, or both. H3 relaxin and insulin produced similar anti-inflammatory effects, and the combination therapy exerted a more obvious impact than the either single therapy (Figure 2B and Supplementary Figure S3A). Moreover, IL-18 and IL-1β in the culture medium were detected by ELISA, which also showed increased concentrations induced by AGE-BSA and decreased concentrations induced by H3 relaxin or/and insulin (Figure 2A). These results indicated that H3 relaxin weakened the process of NLRP3 inflammasome activity in AGE-BSA-treated hRMECs, and these effects were similar to that of insulin.

### H3 Relaxin Suppressed AGE-Induced Apoptosis and Pyroptosis in hRMECs

Transmission electron microscopy was used to observe the ultrastructure of hRMECs to evaluate apoptosis and pyroptosis. We noticed complete cell membranes and nuclear membranes, a rich cytoplasm and well-organized organelles in hRMECs treated with con-BSA. In the AGE-BSA group, a variety of cells in late-stage apoptosis demonstrated a loss of the cytoplasm and reduced cell protuberance. Notably, a few cells exhibited cellular swelling and karyopyknosis, which are the signature features of pyroptosis. After treatment with H3 relaxin, similar to insulin treatment, the cellular morphology was improved with reduced apoptosis, and many cells were in the early or middle stage of apoptosis. hRMECs treated with the combination therapy demonstrated pronounced improvement, and pyroptosis was not observed (Figure 2E). Consistently, western blot analysis showed a decrease in the expression levels of the apoptosis-related proteins, including cleaved caspase-3, cleaved caspase-8 and cleaved caspase-9, and pyroptosis-related proteins, including GSDMD and cleaved caspase-1, in hRMECs treated with H3 relaxin and/or insulin (Figures 2C,F,G and Supplementary Figure SF3B). These results suggested that H3 relaxin played a protective role against apoptosis and pyroptosis in AGE-BSA-induced hRMECs and that the effect of H3 relaxin was similar to that of insulin.

### H3 Relaxin Reduced the AGE-Induced Migration of hRMECs

To determine whether the migration of hRMECs can be induced by AGE-BSA and influenced by H3 relaxin or insulin, the expression of *β*-actin and the migration markers VE-cadherin, ZO-1, occludin, MMP2 and MMP9 were measured. The western blot analysis results revealed that the expression levels of VE-cadherin, ZO-1, occludin, MMP2 and MMP9 were enhanced by AGE-BSA after hRMECs were cultured in the presence of H3 relaxin or insulin, the levels of these proteins were downregulated, and the combination treatment provided stronger protection than that induced by the single treatments (Figure 2D and Supplementary Figure S3C). Wound-healing assays were further used to determine the migration of hRMECs. The data indicated that hRMECs migration was enhanced after stimulation by AGE-BSA, whereas H3 relaxin, insulin or their combination suppressed hRMEC migration (Figures 2H,I). Moreover, the transwell assay results also indicated a decrease in the migration of H3 relaxin- or insulin-treated hRMECs stimulated by AGE-BSA (Figures 2J,K). These data indicated that H3 relaxin prevented the migration of AGE-BSA-stimulated hRMECs, and this protection was similar to that of insulin.

### H3 Relaxin Weakened AGE-Induced Apoptosis and Pyroptosis by Attenuating NLRP3 Inflammasome Activation

**FIGURE 3:**
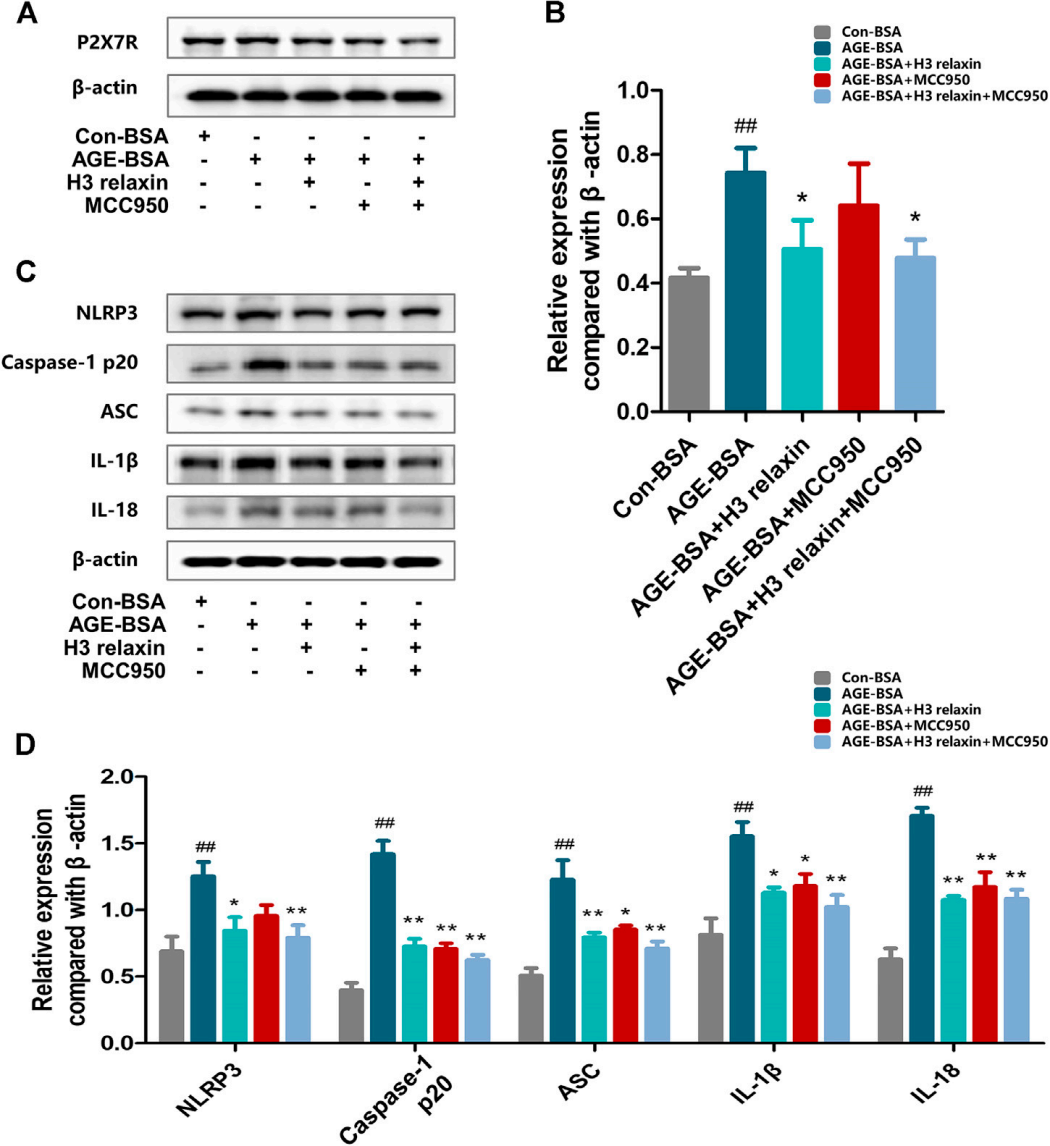
MCC950 and H3 relaxin inhibited AGE-induced inflammatory reaction in hRMECs. **(A,B)** The expression of P2X7R protein were analysed by Western blot. **(C)** The protein expression of NLRP3 inflammasome and inflammatory cytolines were evaluated by western blot after H3 relaxin or/and MCC950 treatment. **(D)** The protein levels of NLRP3 inflammasome and inflammatory cytolines were normalized to *β*-actin. Data are the means ± SD, and each measurement was repeated six times. ^##^ *p* < 0.01 vs. Control group, *p < 0.05 vs. Diabetic group, **p < 0.01 vs. Diabetic group.

**FIGURE 4:**
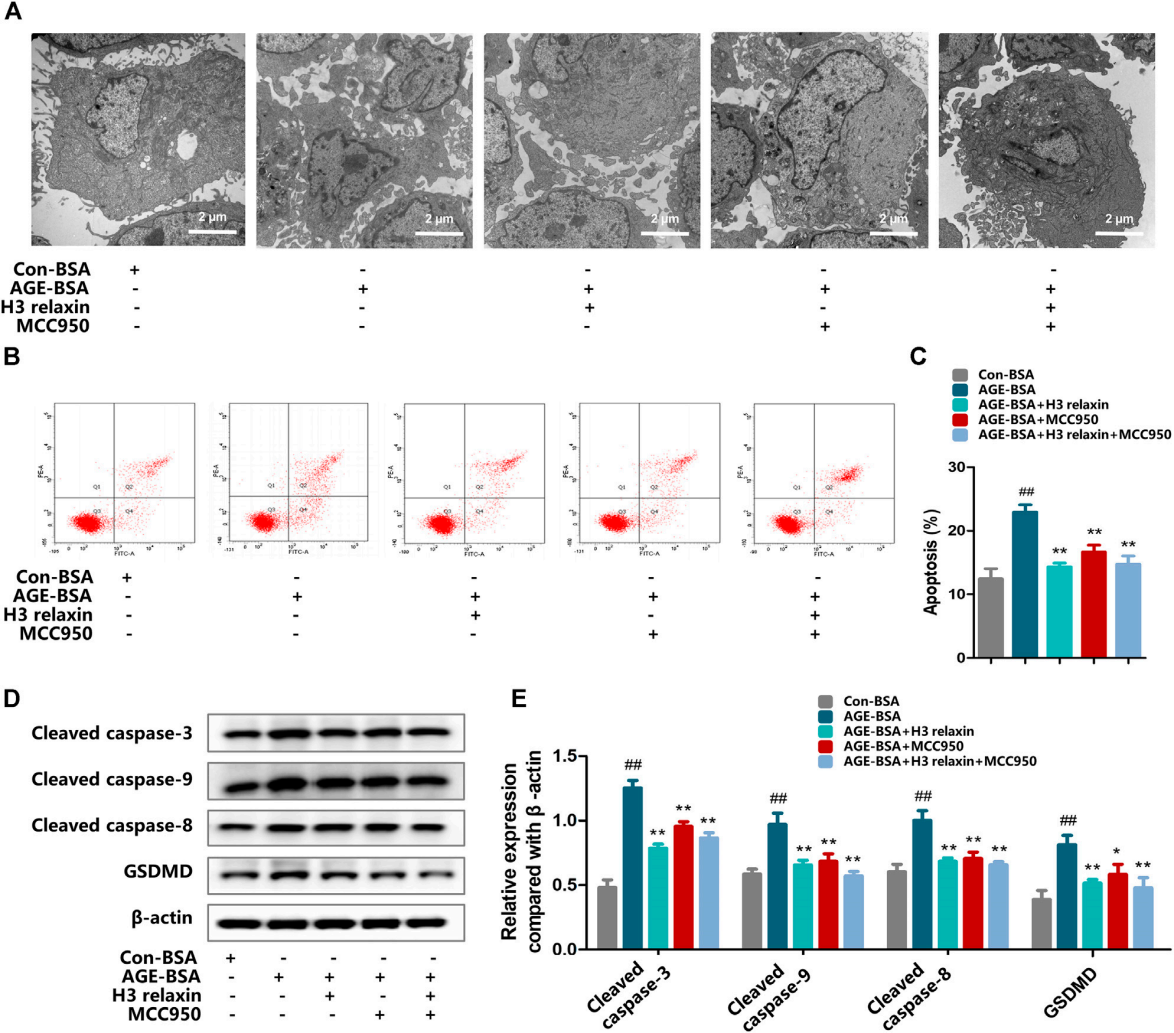
H3 relaxin alleviated AGE-induced apoptosis and pyroptosis in hRMECs by attenuating the activation of NLRP3 inflammasome. **(A)** The apoptosis in hRMECs were observed by transmission electron microscopy (original magnification, ×10,000). Ultrastructural evaluation was performed: After H3 relaxin or/and MCC950 treatment, hRMECs were noticed improvement in apoptosis compared with AGE-BSA group. **(B,C)** Cell apoptosis was measured by flow cytometry. **(D)** The protein expression of cleaved caspase-3, cleaved caspase-9, cleaved caspase-8 and GSDMD were evaluated by western blot. **(E)** The protein levels of cleaved caspase-3, cleaved caspase-9, cleaved caspase-8 and GSDMD were normalized to *β*-actin. Data are the means ± SD, and each measurement was repeated six times. ^##^ *p* < 0.01 vs. Control group, *p < 0.05 vs. Diabetic group, **p < 0.01 vs. Diabetic group.

To determine whether the effect of H3 relaxin on apoptosis and pyroptosis are mediated by the NLRP3 inflammasome, MCC950 was used to decrease the activation of the NLRP3 inflammasome. In the presence of MCC950, the expression levels of cleaved caspase-1, IL-18 and IL-1β were suppressed, whereas the protein levels of NLRP3 and P2X7R were not observably influenced (Figures 3A–D). Proteins related to apoptosis and pyroptosis were detected by western blot analysis, including cleaved caspase-3, cleaved caspase-8, cleaved caspase-9 and GSDMD, the data indicated that the levels of all of these proteins were attenuated by MCC950. In addition, the effect of MCC950 was compared with that of H3 relaxin, and the results indicated that H3 relaxin were somewhat weaker than those of MCC950. Treatment of hRMECs with the combination of H3 relaxin and MCC950 reduced apoptosis and pyroptosis compared with those in hRMECs treated with AGE-BSA, however, the reduction was incomplete (Figures 4A–E). These results indicated that H3 relaxin alleviated AGE-induced apoptosis and pyroptosis and the roles involved in these improvements might be partially mediated by the NLRP3 inflammasome.

### H3 Relaxin Subdued AGE-Induced Migration by Alleviating NLRP3 Inflammasome Activation

**FIGURE 5:**
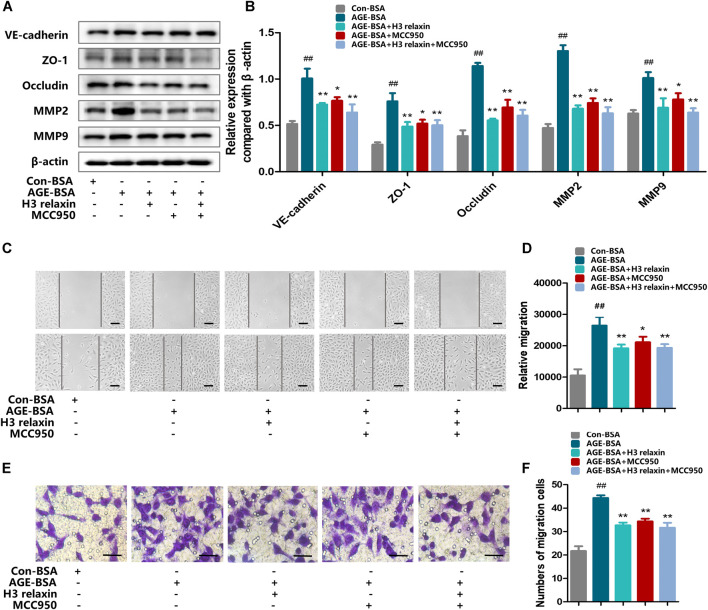
H3 relaxin alleviated AGE-induced migration in hRMECs by attenuating the activation of NLRP3 inflammasome. **(A)** The protein expression of VE-cadherin, ZO-1, occludin, MMP2 and MMP9 were evaluated by western blot. **(B)** The protein levels of VE-cadherin, ZO-1, occludin, MMP2 and MMP9 were normalized to *β*-actin. **(C,D)** Wound-healing assay of hRMECs (Scale bar = 200 µm). **(E,F)** Transwell assay of hRMECs (Scale bar = 200 µm). Data are the means ± SD, and each measurement was repeated six times. ^##^ *p* < 0.01 vs. Control group, *p < 0.05 vs. Diabetic group, **p < 0.01 vs. Diabetic group.

We further evaluated the effect of H3 relaxin on migration by inhibiting the NLRP3 inflammasome. The expression levels of proteins related to migration, including VE-cadherin, ZO-1, occludin, MMP2 and MMP9 were assayed. The results showed that hRMECs treated with MCC950 manifested a decrease in the levels of VE-cadherin, ZO-1, occludin, MMP2 and MMP9, accompanied by reduced expression levels of cleaved caspase-1, IL-18 and IL-1β. hRMECs treated with H3 relaxin also exhibited improvements in VE-cadherin, ZO-1, occludin, MMP2 and MMP9; unlike the MCC950 group, the H3 relaxin group showed improvements in all components of the NLRP3 inflammasome. Treatment with MCC950 and H3 relaxin decreased the levels of proteins related to the migration and NLRP3 inflammasome compared with those in cells treated with AGE-BSA (Figures 5A,B). The wound-healing and transwell assays showed a similar trend in migration (Figures 5C–F). These data suggested that activation of the NLRP3 inflammasome may contribute to H3 relaxin-induced suppression of AGE-stimulated migration.

### H3 Relaxin Suppressed AGE-Induced Retinal Apoptosis and Pyroptosis by Attenuating P2X7R-Mediated NLRP3 Inflammasome Activation

**FIGURE 6:**
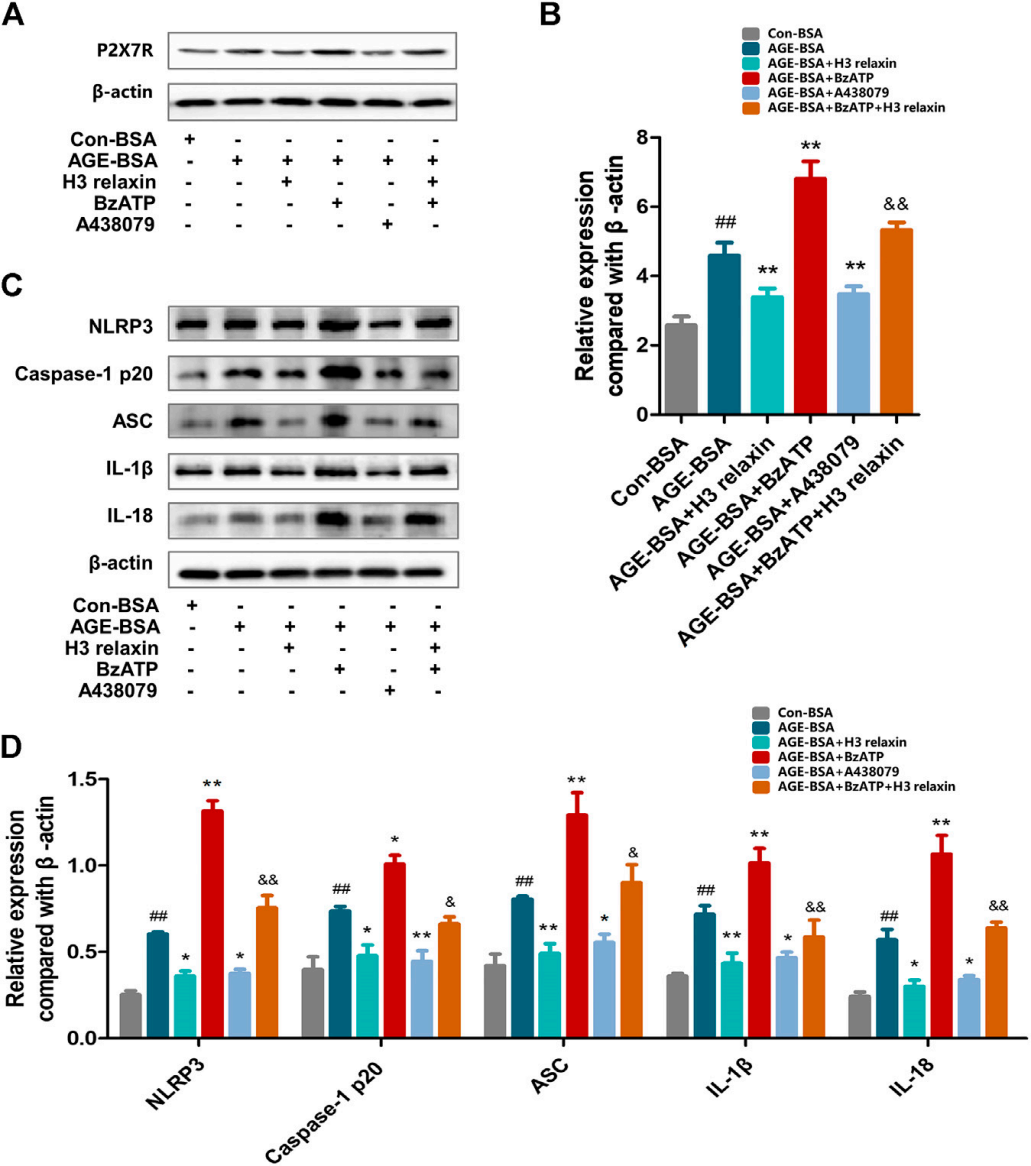
The regulation of P2X7R changed the inflammatory reaction induced by AGE-BSA in hRMECs. **(A,B)** The expression of P2X7R protein were analysed by Western blot. **(C)** The protein expression of NLRP3 inflammasome and inflammatory cytolines were evaluated by western blot after P2X7R agonist or inhibitor treatment. **(D)** The protein levels of NLRP3 inflammasome and inflammatory cytolines were normalized to *β*-actin. Data are the means ± SD, and each measurement was repeated six times. ##*p* < 0.01 vs. Control group, *p < 0.05 vs. Diabetic group, **p < 0.01 vs. Diabetic group. ^&^ *p* < 0.05 vs. AGE-BSA+BzATP group, ^&&^ *p* < 0.01 vs. AGE-BSA+BzATP group.

**FIGURE 7:**
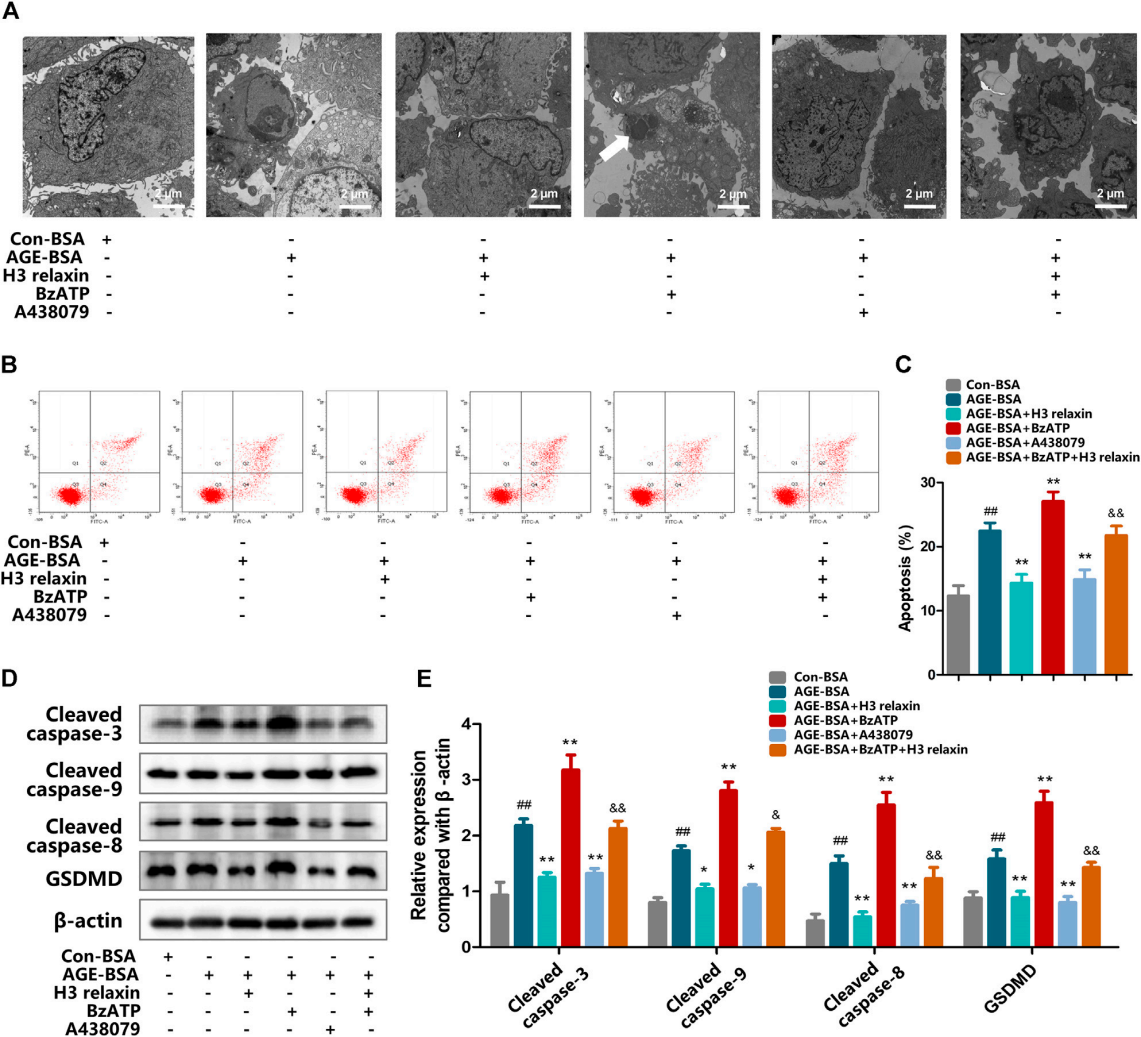
The regulation of P2X7R changed apoptosis and pyroptosis induced by AGE-BSA in hRMECs. **(A)** The apoptosis in hRMECs were observed by transmission electron microscopy. (original magnification, ×10,000). Ultrastructural evaluation was performed: After H3 relaxin or/and A438079 treatment, hRMECs were noticed improvement in apoptosis compared with AGE-BSA group. BzATP enhanced cell injury into late phase of apoptosis and a phagosome can be noticed (arrow). **(B,C)** Cell apoptosis was measured by flow cytometry. **(D)** The protein expression of cleaved caspase-3, cleaved caspase-9, cleaved caspase-8 and GSDMD were evaluated by western blot. **(E)** The protein levels of cleaved caspase-3, cleaved caspase-9, cleaved caspase-8 and GSDMD were normalized to *β*-actin. Data are the means ± SD, and each measurement was repeated six times. ^##^ *p* < 0.01 vs. Control group, *p < 0.05 vs. Diabetic group, **p < 0.01 vs. Diabetic group. ^&^ *p* < 0.05 vs. AGE-BSA+BzATP group, ^&&^ *p* < 0.01 vs. AGE-BSA+BzATP group.

ATP-mediated K+ efflux through P2X7R is an upstream signal essential for the activation of the NLRP3 inflammasome, which increases the secretion of IL-1β and IL-18 (Surprenant et al., 1996). Our team found that H3 relaxin reduced the expression of P2X7R induced by AGE-BSA (Figure 6A–D). Then, the potential mechanism of the H3 relaxin-dependent reduction in AGE-induced apoptosis and pyroptosis via P2X7R-mediated activation of the NLRP3 inflammasome was investigated. Initially, hRMECs were treated with AGE-BSA and the P2X7R agonist BzATP or the P2X7R inhibitor A438079. The BzATP treatment enhanced the expression of the NLRP3 inflammasome and proteins related to apoptosis and pyroptosis, including cleaved caspase-3, cleaved caspase-8 and cleaved caspase-9 and GSDMD, whereas the treatment with A438079 weakened the expression levels of these proteins, similar to the results obtained in the H3 relaxin treatment group. Treatment with a combination of H3 relaxin and BzATP decreased the levels of these proteins compared with those in cells treated with BzATP alone. The IL-18 and IL-1β levels in the culture medium followed a similar trend (Figures 7A–E). These data indicated that H3 relaxin might play roles in AGE-induced apoptosis and pyroptosis, which are at least partially mediated by P2X7R-dependent activation of the NLRP3 inflammasome.

### H3 Relaxin Mitigated AGE-Induced Retinal Cell Migration by Alleviating P2X7R-Mediated NLRP3 Inflammasome Activation

**FIGURE 8:**
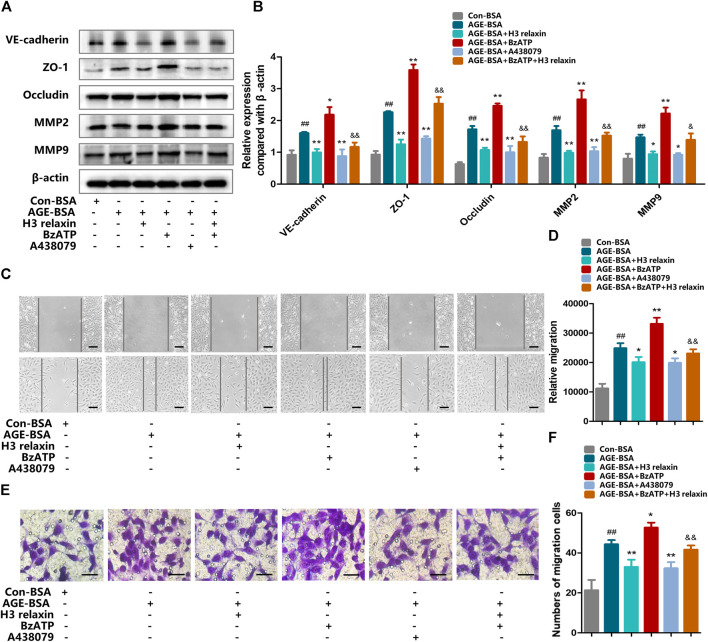
The regulation of P2X7R changed migration induced by AGE-BSA in hRMECs. **(A)** The protein expression of VE-cadherin, ZO-1, occludin, MMP2 and MMP9 were evaluated by western blot. **(B)** The protein levels of VE-cadherin, ZO-1, occludin, MMP2 and MMP9 were normalized to *β*-actin. **(C,D)** Wound-healing assay of hRMECs (Scale bar = 200 µm). **(E,F)** Transwell assay of hRMECs (Scale bar = 200 µm). Data are the means ± SD, and each measurement was repeated six times. ^##^ *p* < 0.01 vs. Control group, *p < 0.05 vs. Diabetic group, **p < 0.01 vs. Diabetic group. ^&^ *p* < 0.05 vs. AGE-BSA+BzATP group, ^&&^ *p* < 0.01 vs. AGE-BSA+BzATP group.

We further investigated whether H3 relaxin reduces AGE-induced migration via P2X7R-mediated NLRP3 inflammasome activation. Compared with con-BSA, H3 relaxin and A438079 both reduced the expression of proteins related to migration, and BzATP increased the migration of hRMECs induced by AGE-BSA, which was consistent with the trend for NLRP3-mediated inflammation (Figures 8A,B). This observation was further supported by wound-healing and transwell assays (Figures 8C–F). These data indicated that H3 relaxin might attenuate AGE-induced migration, which at least played a protective role partially through P2X7R-mediated NLRP3 inflammasome activation.

## Discussion

Studies have shown that the levels of caspase-1 and the inflammatory cytokine IL-18 are increased in patients with proliferative diabetic retinopathy (PDR) and the level of NLRP3 in the optic disc in the eyes of patients with PDR is higher than that in the eyes of patients with inactive neovascularization (Qi et al., 2014). Inhibition of the NLRP3 inflammasome enhances insulin sensitivity and glucose tolerance compared with no treatment (Vandanmagsar et al., 2011). Here, we observed that high glucose increased the expression of the NLRP3 inflammasome in the rat retina. In AGE-BSA-induced hRMECs, the levels of the NLRP3 inflammasome and its effector cytokines were also significantly increased. The detection of IL-18 and IL-1β in rat plasma and culture medium supported the conclusion. Recently, a diaryl sulfonylurea compound, MCC550, was reported to be a highly specific inhibitor of NLRP3-mediated inflammation, and this compound was initially identified the most effective inhibitor of the IL-1β-releasing enzyme (Perregaux et al., 2001). Our results showed that MCC950 controlled the activation of the NLRP3 inflammasome by inhibiting cleaved caspase-1 and decreasing the levels of the downstream cytokines IL-1β and IL-18, thus reducing the migration, apoptosis and pyroptosis of hRMECs by inhibiting the production of cleaved caspase-1 and inflammatory cytokines. However, MCC950 did not influence the expression of NLRP3 or caspase-1 precursors. MCC950 played an anti-inflammatory role in the pathogenesis of glucose-mediated retinopathy, and our data are consistent with the results of previous reports.

P2X7R is an oxidative stress and metabolic sensor that contributes to the activation of the NLRP3 inflammasome as an activator. In the retinas of murine and primates, P2X7R is present in the retinal microvascular system, neural cells and macrophages. On the condition of low extracellular ATP production, phagocytosis contributes to cleaning cell debris, when the concentrations of ATP are high, neuroinflammation enhanced instead of phagocytosis (Ho et al., 2016). After binding with ATP, P2X7R forms a nonselective cation channel on the cell surface (Kahlenberg and Dubyak, 2004). It is worth noting that the intimate relationship between P2X7R and NLRP3 inflammasome shows significant contribution to the activation of inflammasome. Firstly, P2X7R can assist NLRP3 to precisely focus on the position of K^+^ efflux and Ca^2+^ influx. Moreover, P2X7R recruitment also amplifies proinflammatory signals by regulating the expression levels of NLRP3 (Franceschini et al., 2015). Previous studies have demonstrated that a deficiency in P2X7R can provide resistance to the induction of diabetes and have suggested that P2X7R-targeted therapy of clinical diabetes may be feasible. P2X7R mediates the accumulation of the NLRP3 inflammasome and is involved the development of diabetic cardiomyopathy (Zhang et al., 2018). Our findings are consistent with previous studies that have demonstrated that enhancement of P2X7R signaling by the agonist BzATP can significantly activate the NLRP3 inflammasome stimulated by AGE-BSA and that the inhibitor A438079 attenuates the inflammatory response. Our data indicate that P2X7R indirectly affected AGE-induced hRMECs via the NLRP3 inflammasome. An increase in the expression of P2X7R changed the interendothelial cell junctions composed of protein complexes, including tight junctions and adherens junctions which contain Vascular endothelial (VE)-cadherin and zonula occludens (ZO)-1 respectively. In addition, upregulation of the expression of highly conserved MMPs, which are involved in extracellular matrix remodeling, angiogenesis, cell migration and proliferation, enhanced hRMECs migration and influenced the steady state of retinal microvessels (Firth et al., 1983). Moreover, our data indicated an increase in pyroptosis in hRMECs, which was consistent with the occurrence of apoptosis. Activation of the NLRP3 inflammasome in multiple pathological states can be induced by the recruitment of pro-IL-1β and pro-IL-18, which are cleaved by caspase-1, leading to pore formation, osmotic swelling, early loss of membrane integrity and eventual rapid inflammatory lytic death (Miao et al., 2011). Studies have proven that cell apoptosis and migration are closely related. Endothelial cell apoptosis plays an indispensable role in the development and angiogenesis of the vascular system and is strictly regulated by the expression of proapoptotic and antiapoptotic factors. Recently, knockout of bcl-2 was reported to influence the proliferation, adhesion, migration and capillary morphogenesis of hRMECs, which destroyed the relationship between cell survival and angiogenesis-promoting signals, prevented the formation of capillary morphology, decreased the branching and formation of retinal vessels, and reduced the numbers of endothelial cells and pericytes. (Kondo et al., 2008).

Relaxin is a peptide hormone composed of 57 amino acids with a structure similar to that of insulin (Bathgate et al., 2006). Bitto and colleagues noticed that relaxin significantly improved the wound-healing process in obese diabetic mice and effectively induced vascular regeneration by improving the expression of damaged endothelial growth factor (Bonner et al., 2013). A Russian study suggested that relaxin may act as a substitute when insulin is less effective as the receptors of relaxin are expressed in the pancreas, liver and muscle, which is consistent with the distribution of target organs of insulin action (Whittaker et al., 2003; Halls et al., 2007). Previous studies have shown that H3 relaxin plays an important role in diabetic vascular complications. The antifibrotic effect of H3 relaxin in diabetic cardiomyopathy has been confirmed, as has its beneficial effect on diabetic nephropathy (Zhang et al., 2018). Our results focus on another type of diabetic microvascular complication, diabetic retinopathy. In animal models, 14 days of continuous treatment with H3 relaxin significantly reduced retinal inflammation and apoptosis, attenuated the thickening of the basement membrane of the retinal capillary wall, weakened the swelling of endothelial cells and pericytes, and improved the morphology of cellular organelles. The photoreceptors on retinas are sensitive to high concentration of glucose, and the structural modifications precede the vasculopathy in the retinas. (Maisto et al., 2020) In our study, we noticed denatured mitochondria especially in the outer segments of the photoreceptors, as well as at the periphery of cone and rod bodies in the retinas of diabetic mice, and after H3 relaxin or/and treatment, the structural modifications of mitochondria were mildly but not totally improved. It is also reported that the mitochondria were the worst victims among organelles due to the changes in the mitochondrial fusion fission and transport proteins In the retina of diabetic mice (Zhong & Kowluru, 2011; Platania et al., 2019). *In vitro* experiments demonstrated that H3 relaxin attenuated the expression of the NLRP3 inflammasome in hRMECs stimulated by AGE-BSA and also inhibited the migration, apoptosis and pyroptosis of cells. AGE-BSA at a concentration of 100 µg/ml has little impact on the viability of hRMECs but influences the expression of proteins related to apoptosis and pyroptosis. Images acquired by electron microscopy also showed that the majority of AGE-BSA-induced hRMECs were in the middle stage of apoptosis, with some cells in the late stage of apoptosis. After H3 relaxin treatment, the majority of hRMECs were in the early stage of apoptosis, and pyroptosis was not observed.

MCC950 treatment of hRMECs alleviated the expression of NLRP3 inflammasome, and the migration, apoptosis and pyroptosis were synchronously suppressed. Treatment of hRMECs with a combination of MCC950 and H3 relaxin mildly reduced the expression of proteins related to migration, apoptosis and pyroptosis compared with those in cells treated with the agents separately, however, the mitigation was not complete. This phenomenon may be due to the intermediate role of the NLRP3 inflammasome in the antiapoptotic and antimigratory effects of H3 relaxin; when the NLRP3 inflammasome is partially inhibited, the beneficial effects of H3 relaxin are partially affected and are not completely eliminated. Therefore, treatment with a combination of MCC950 and H3 relaxin did not produce enough synergistic effects.

Our subsequent experiments focused on P2X7R, the upstream signal of the NLRP3 inflammasome. Studies have showed different opinions on how P2X7R contribute to retinal microvascular damage induced by high glucose through inflammatory signals. It is likely supposed that the protective mechanism of P2X7R antagonist are attributed to the inhibition of both infammatory cytokine and VEGF release, resulting in enhanced angiogenesis and vascular permeability, which trigger to the invasion of DR (Clapp et al., 2019). Sugiyama et al. suggested that the activation of P2X7R can increase the vulnerability of retinal microvessels by formating large nonselective transmembrane pores to drive cation fluxes and intensive cell apoptosis in retinal microvascular cells (Sugiyama et al., 2004). However, in subsequent studies, the authors suggested an alternative mechanism. ATP-P2X7R may kill the cells via voltage-dependent Ca^2+^ channels (VDCC) because pore formation may be decreased by ATP activation of P2Y4R, which still need further discuss (Sugiyama et al., 2005). Our results indicated that AGE-BSA triggered the activation of P2X7R, followed by the activation of the NLRP3 inflammasome in hRMECs. H3 relaxin inhibited the activation of P2X7R induced by AGE-BSA, suggesting that H3 relaxin regulated the activation of the NLRP3 inflammasome through P2X7R under AGE-BSA-stimulated conditions. We found that the inhibition of P2X7R significantly reduced the expression and activation of the NLRP3 inflammasome induced by AGE-BSA, indicating that the initiation of NLRP3 inflammasome activation is dependent on the activation of P2X7R mediated by AGE-BSA. AGE-BSA promoted NLRP3 inflammasome-dependent migration, apoptosis and pyroptosis in hRMECs via the P2X7R pathway. Therefore, we elucidated a novel mechanism of inhibition of P2X7R-mediated activation of the NLRP3 inflammasome by H3 relaxin in the pathogenesis of diabetic retinopathy.

## Conclusion

In summary, our results illustrated a new mechanism of H3 relaxin-dependent inhibition of retinal injury induced by AGE-BSA via P2X7R-mediated activation of the NLRP3 inflammasome in hRMECs. Therefore, our findings provide new insights into the mechanisms involved in the migration, apoptosis and pyroptosis of hRMECs and demonstrate that H3 relaxin is a potential therapeutic agent for ameliorating the activation of the NLRP3 inflammasome associated with high glucose, especially that in AGE-BSA-induced diabetic retinopathy.

## Data Availability Statement

The original contributions presented in the study are included in the article/Supplementary Material, further inquiries can be directed to the corresponding author.

## Ethics Statement

The animal study was reviewed and approved by the Ethics Committee of The First Affiliated Hospital of Harbin Medical University.

## Author Contributions

KY, JL, XZ, and HK designed the study. KY, JL, and XZ refined the process. KY, JL, XZ, and ZR participated in rats section. KY, JL, ZR, LG, and YW participated *in vitro* experiment. KY and JL performed most of the experiments. ZR, LG, YW, WL, XM, and MH performed some of the experiments. KY, WL, XM, and MH analyzed the data. KY wrote the manuscript. HK supervised the study and the manuscript. All authors reviewed and approved the final draft.

## Funding

This work was supported by the National Natural Science Foundation of China (No. 81670739) and the Research innovation foundation of Harbin medical university (No. YJSKYCX2019-34HYD).

## Conflict of Interest

The authors declare that the research was conducted in the absence of any commercial or financial relationships that could be construed as a potential conflict of interest.
