# Supplementary figures and images for: H3 Relaxin Alleviates Migration, Apoptosis and Pyroptosis Through P2X7R-Mediated Nucleotide Binding Oligomerization Domain-Like Receptor Protein 3 Inflammasome Activation in Retinopathy Induced by Hyperglycemia
Source: Front Pharmacol. 2020 Dec 16;11:603689. doi: 10.3389/fphar.2020.603689 (PMC7873867; doi:10.3389/fphar.2020.603689)

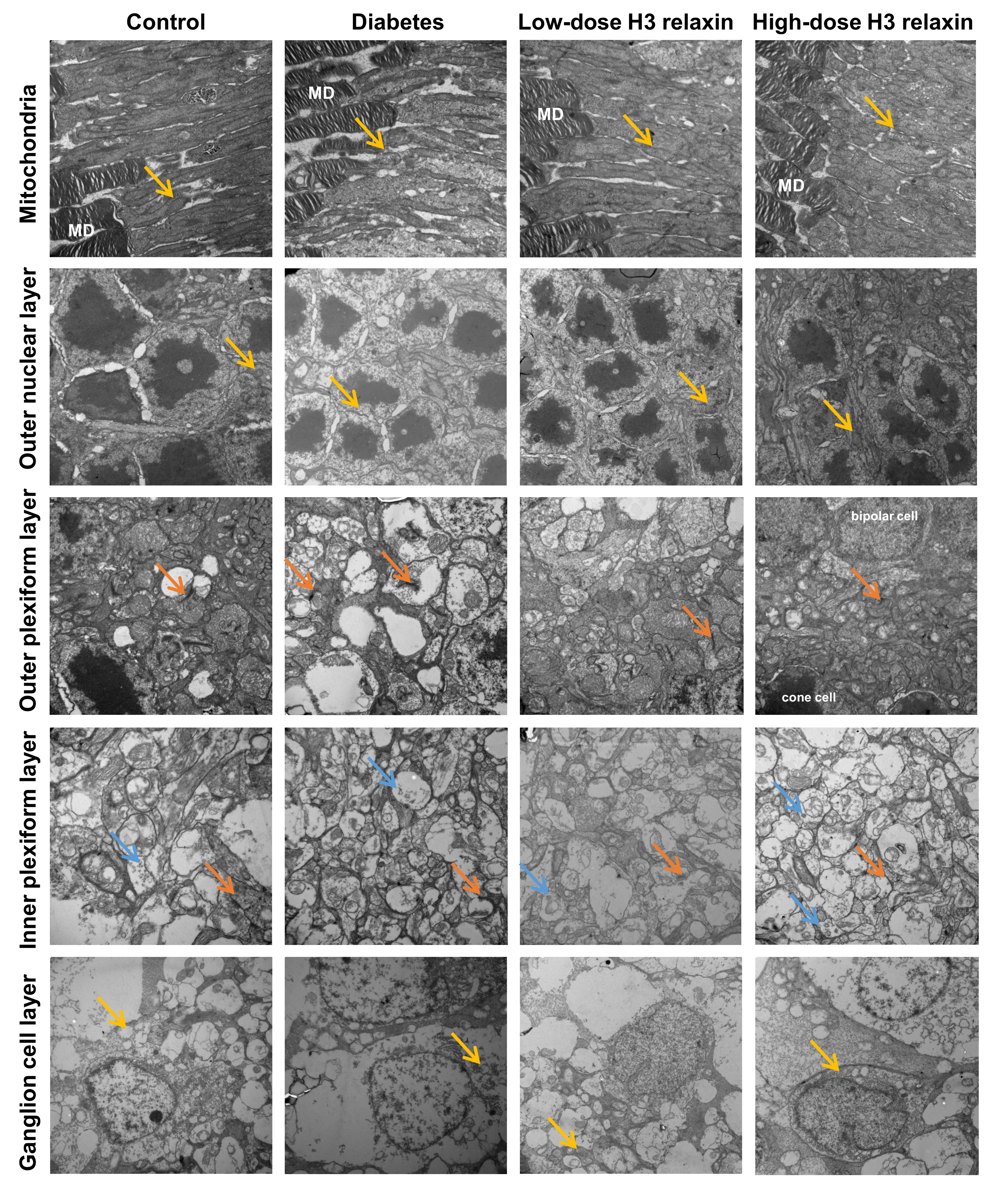

Supplement: Supplementary file 1 [file image1.tif]

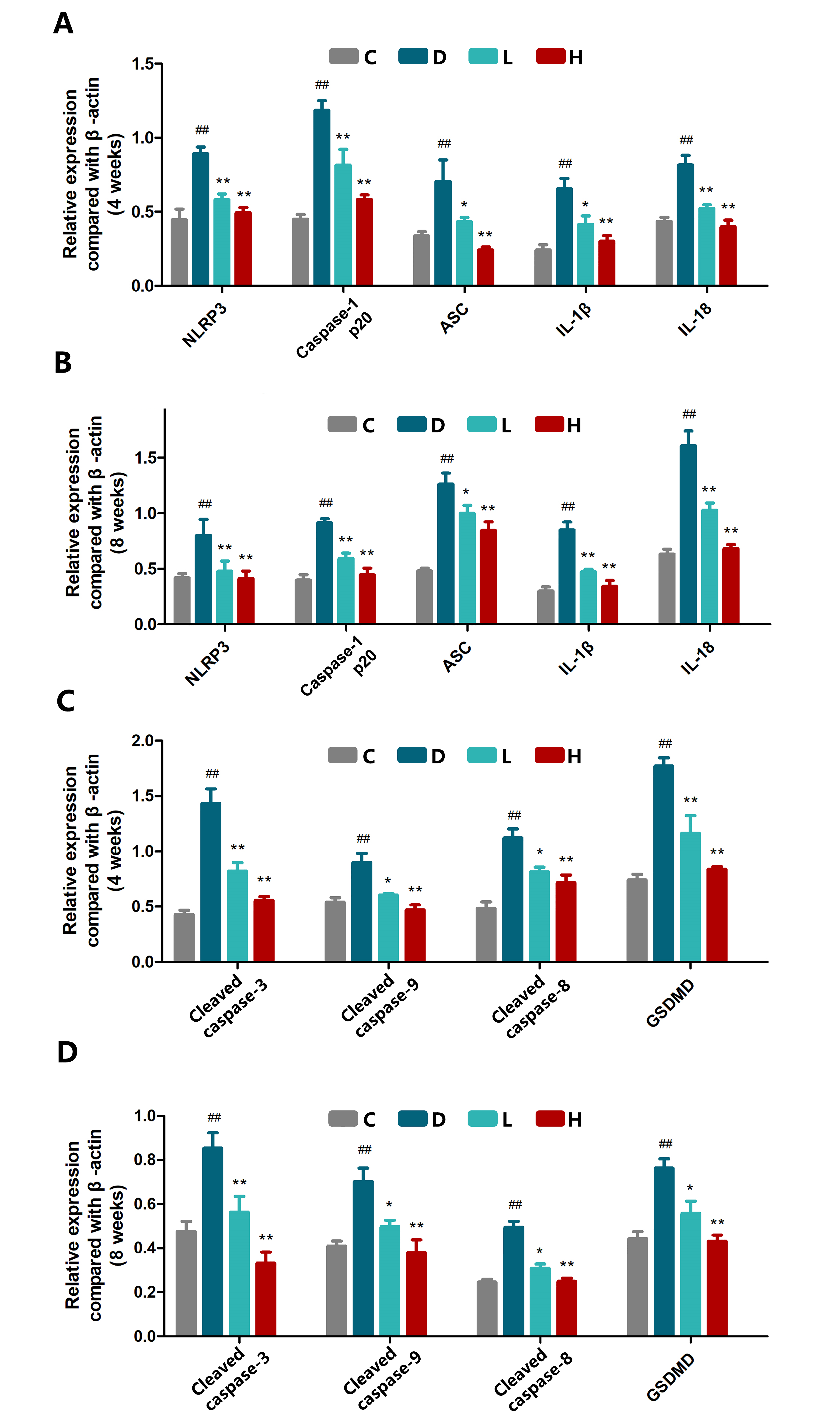

Supplement: Supplementary file 2 [file image2.tif]

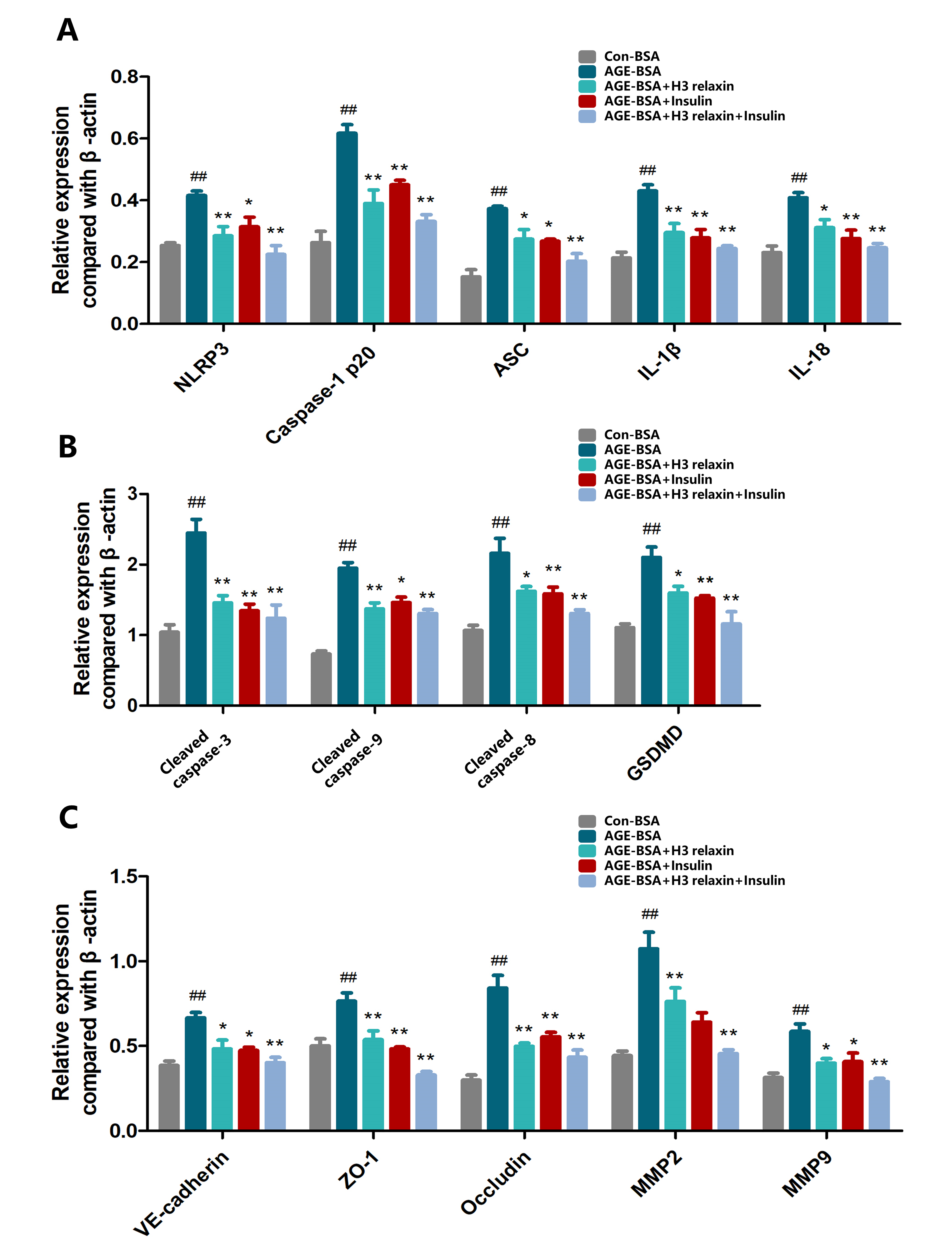

Supplement: Supplementary file 3 [file image3.tif]
